# Supplementary material for: Cerebrospinal fluid tau, neurogranin, and neurofilament light in Alzheimer's disease
Source: EMBO Mol Med. 2016 Aug 17;8(10):1184–96. doi: 10.15252/emmm.201606540 (PMC5048367; doi:10.15252/emmm.201606540)
Supplement: Supplementary file 1 — Expanded View Figures PDF [file EMMM-8-1184-s001.pdf]

Expanded View Figures

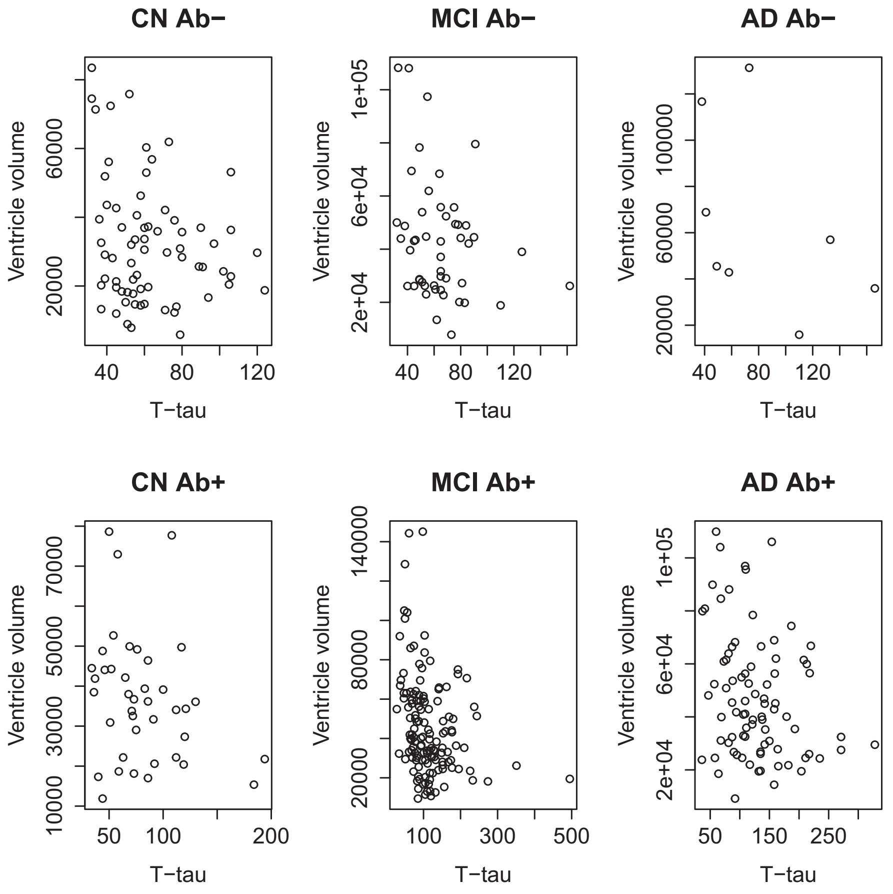

Figure EV1. CSF T-tau and ventricular volume.
Scatter plots showing CSF T-tau (ng/l) and ventricular volumes (ml) in different diagnostic group, separately for Aβ-negative (upper row) and Aβ-positive subjects (lower row). The plots illustrate the general observation that very high CSF T-tau levels are often seen in subjects with relatively small ventricles, independent of diagnostic group and Aβ status.

 © 2016 The Authors

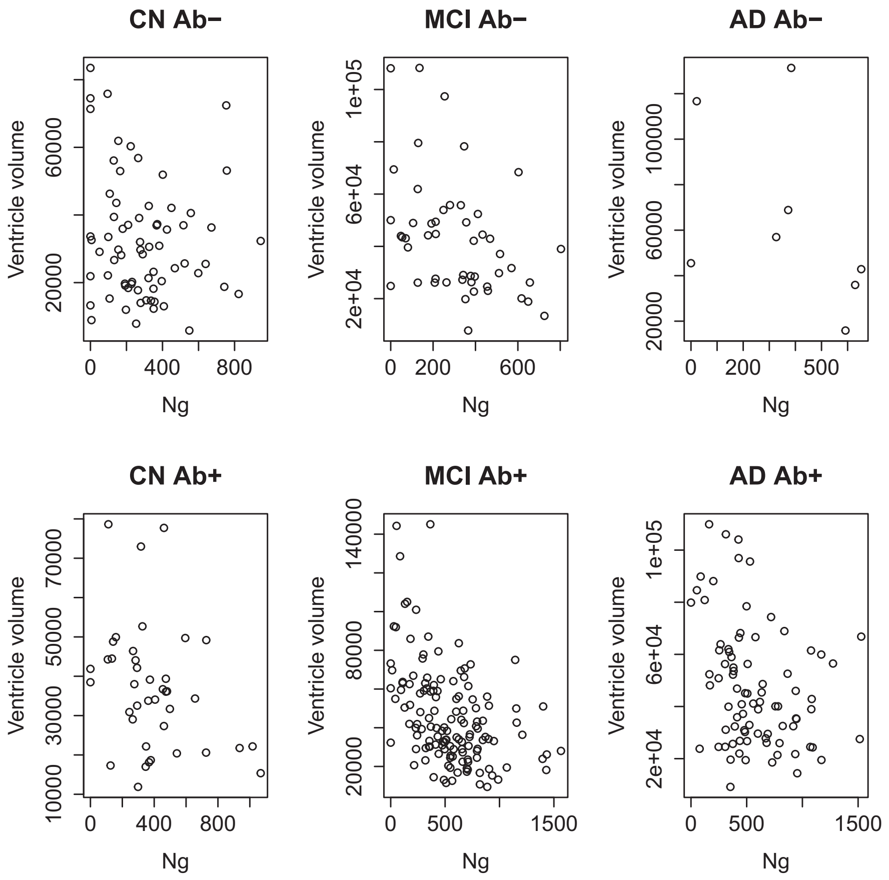

Figure EV2. CSF Ng and ventricular volume.
Scatter plots showing CSF Ng (ng/l) and ventricular volumes (ml) in different diagnostic group, separately for Aβ-negative (upper row) and Aβ-positive subjects (lower row). The plots illustrate the general observation that very high CSF Ng levels are often seen in subjects with relatively small ventricles, independent of diagnostic group and Aβ status.
