# Supplementary material for: Cerebrospinal fluid tau, neurogranin, and neurofilament light in Alzheimer's disease
Source: EMBO Mol Med. 2016 Aug 17;8(10):1184–96. doi: 10.15252/emmm.201606540 (PMC5048367; doi:10.15252/emmm.201606540)
Supplement: Supplementary file 2 — Table EV1 [file EMMM-8-1184-s002.docx]

**Table EV1**

**Diagnostic accuracy of CSF biomarkers when adjusting for covariates**

| **Group** | **Covariates** | **Model** | **T-tau** | **Ng** | **NFL** | **AUC** | **AIC** |
| --- | --- | --- | --- | --- | --- | --- | --- |
| AD vs CN | Age, sex, education | T-tau only | **1.65 (<.001)** |  |  | 82.4^b,e,g^ (77.2-88.3) | 216.9 |
|  |  | Ng only |  | **.83 (<.001)** |  | 71.3^a,c-g^ (65.3-79.4) | 257.5 |
|  |  | NFL only |  |  | **1.98 (<.001)** | 84.1^b,e,g^ (79.5-89.7) | 207.6 |
|  |  | T-tau & Ng | **2.19 (<.001)** | **-.63 (.047)** |  | 83.3^b,e,g^ (78.3-89.5) | 214.7 |
|  |  | T-tau & NFL | **1.17 (<.001)** |  | **1.50 (<.001)** | 87.5^a-d,f^ (83.3-92.4) | 189.1 |
|  |  | Ng & NFL |  | .39 (.064) | **1.81 (<.001)** | 84.7^b,e,g^ (80.4-90.4) | 206.0 |
|  |  | T-tau & Ng & NFL | **1.78 (<.001)** | **-.71 (.040)** | **1.54 (<.001)** | 88.1^a-d,f^ (84.5-93.4) | 186.6 |
|  |  | Only covariates |  |  |  | 60.5^a-g^ (48.6-69.1) | 281.1 |
|  | Age, sex, education, CSF Aβ42 | T-tau only | **1.20 (<.001)** |  |  | 87.8^b,e,f,g^ (83-4-92.8) | 192.0 |
|  |  | Ng only |  | **.56 (.0063)** |  | 83.5^a,c-g^ (79.0-89.7) | 211.8 |
|  |  | NFL only |  |  | **1.82 (<.001)** | 90.8^b^ (87.1-95.0) | 166.5 |
|  |  | T-tau & Ng | **1.72 (<.001)** | -.62 (.075) |  | 88.2^b,e,g^ (83.9-93.4) | 190.6 |
|  |  | T-tau & NFL | **.62 (.041)** |  | **1.55 (<.001)** | 91.5^a,b,d^ (88.1-95.6) | 163.9 |
|  |  | Ng & NFL |  | .098 (.69) | **1.78 (<.001)** | 90.8^a,b^ (87.5-95.1) | 168.4 |
|  |  | T-tau & Ng & NFL | **1.10 (.012)** | -.59 (.11) | **1.56 (<.001)** | 91.8^a,b,d^ (88.7-95.7) | 163.2 |
|  |  | Only covariates |  |  |  | 81.1 (75.9-88.3) | 218.1 |
| PMCI vs SMCI | Age, sex, education | T-tau only | **.56 (.0040)** |  |  | 68.0 (58.3-76.8) | 224.8 |
|  |  | Ng only |  | **.42 (.025)** |  | 62.2 (55.7-72.6) | 229.1 |
|  |  | NFL only |  |  | .34 (.068) | 59.6^g^ (52.2-70.1) | 230.9 |
|  |  | T-tau & Ng | **.58 (.047)** | -.019 (.95) |  | 68.1 (59.1-77.5) | 226.8 |
|  |  | T-tau & NFL | **.51 (.011)** |  | .20 (.28) | 67.1 (59.3-77.2) | 225.6 |
|  |  | Ng & NFL |  | **.38 (.041)** | .29 (.11) | 62.8 (58.2-74.1) | 228.4 |
|  |  | T-tau & Ng & NFL | .49 (.10) | .023 (.94) | .20 (.28) | 67.2^c^ (60.2-77.6) | 227.6 |
|  |  | Only covariates |  |  |  | 53.6^a,d-g^ (46.1-65.4) | 232.5 |
|  | Age, sex, education, CSF Aβ42 | T-tau only | .26 (.21) |  |  | 69.7 (63.5-80.4) | 214.7 |
|  |  | Ng only |  | .17 (.39) |  | 68.6 (62.8-79.7) | 215.5 |
|  |  | NFL only |  |  | .029 (.12) | 69.0 (62.7-79.4) | 213.8 |
|  |  | T-tau & Ng | .27 (.37) | -.013 (.96) |  | 69.7 (64.3-80.8) | 216.7 |
|  |  | T-tau & NFL | .19 (.38) |  | .24 (.20) | 69.9 (64.3-81.0) | 215.0 |
|  |  | Ng & NFL |  | .15 (.45) | .28 (.13) | 70.1 (63.9-80.7) | 215.2 |
|  |  | T-tau & Ng & NFL | .15 (.63) | .048 (.87) | .25 (.20) | 70.0 (65.1-81.3) | 217.0 |
|  |  | Only covariates |  |  |  | 67.1^g^ (60.9-78.0) | 214.3 |

A separate logistic regression model was fit for each combination of neurodegeneration biomarkers in AD dementia versus CN and in PMCI versus SMCI. The table includes coefficients (log odds) with p-values from each predictor, AUC, and AIC. For AUC, the letters a-g indicate significant differences (p < .05) versus other models: T-tau (a), Ng (b), NFL (c), T-tau & Ng (d), T-tau & NFL (e), Ng & NFL (f), and T-tau & Ng & NFL (g).
